# Determinants of treatment-related paradoxical reactions during anti-tuberculosis therapy: a case control study

**DOI:** 10.1186/s12879-016-1816-4

**Published:** 2016-09-06

**Authors:** Colin Stewart Brown, Colette Joanne Smith, Ronan Angus MacCormick Breen, Lawrence Peter Ormerod, Rahul Mittal, Marie Fisk, Heather June Milburn, Nicholas Martin Price, Graham Henry Bothamley, Marc Caeroos Isaac Lipman

**Affiliations:** 1 Hospital for Tropical Diseases, University College London Hospitals Foundation Trust, 235 Euston Road, London, NW1 2BU UK; 2 UCL Division of Infection and Immunity, University College London, Rowland Hill Street, London, NW3 2PF UK; 3 Royal Free Campus, University College London, Rowland Hill Street, London, NW3 2PF UK; 4 Guy’s and St Thomas’ Hospital NHS Trust, Westminster Bridge Road, London, SE1 7EH UK; 5 Royal Blackburn Hospital, Blackburn, Lancs BB2 3LR UK; 6 Royal Free London NHS Foundation Trust, Pond Street, London, NW3 2QG UK; 7 Homerton University Hospital, Homerton Row, London, E9 6SR UK; 8 UCL Respiratory, Division of Medicine, University College London, Rowland Hill Street, London, NW3 2PF UK

**Keywords:** Tuberculosis, Treatment, Paradoxical reactions, HIV, IRIS, Predictors, Determinants, Ethnicity

## Abstract

**Background:**

Inflammatory response following initial improvement with anti-tuberculosis (TB) treatment has been termed a paradoxical reaction (PR). HIV co-infection is a recognised risk, yet little is known about other predictors of PR, although some biochemical markers have appeared predictive. We report our findings in an ethnically diverse population of HIV-infected and uninfected adults.

**Methods:**

Prospective and retrospective clinical and laboratory data were collected on TB patients seen between January 1999–December 2008 at four UK centres selected to represent a wide ethnic and socio-economic mix of TB patients. Data on ethnicity and HIV status were obtained for all individuals. The associations between other potential risk factors and PR were assessed in a nested case-control study. All PR cases were matched two-to-one to controls by calendar time and centre.

**Results:**

Of 1817 TB patients, 82 (4.5 %, 95 % CI 3.6–5.5 %) were identified as having a PR event. The frequency of PR was 14.4 % (18/125; 95 % CI 8.2–20.6 %) and 3.8 % (64/1692; 2.9–4.7) for HIV-positive and HIV-negative individuals respectively. There were no differences observed in PR frequency according to ethnicity, although the site was more likely to be pulmonary in those of black and white ethnicity, and lymph node disease in those of Asian ethnicity. In multivariate analysis of the case-control cohort, HIV-positive patients had five times the odds of developing PR (aOR = 5.05; 95 % CI 1.28–19.85, *p* = 0.028), whilst other immunosuppression e.g. diabetes, significantly reduced the odds of PR (aOR = 0.01; 0.00–0.27, *p* = 0.002). Patients with positive TB culture had higher odds of developing PR (aOR = 6.87; 1.31–36.04, *p* = 0.045) compared to those with a negative culture or those in whom no material was sent for culture. Peripheral lymph node disease increased the odds of a PR over 60-fold 4(9.60–431.25, *p* < 0.001).

**Conclusion:**

HIV was strongly associated with PR. The increased potential for PR in people with culture positive TB suggests that host mycobacterial burden might be relevant. The increased risk with TB lymphadenitis may in part arise from the visibility of clinical signs at this site. Non-HIV immunosuppression may have a protective effect. This study highlights the difficulties in predicting PR using routinely available demographic details, clinical symptoms or biochemical markers.

## Background

Inflammatory response associated with treatment for tuberculosis (TB) has been termed a paradoxical reaction. This is defined as a worsening of clinical or radiological findings following the initiation of appropriate anti-tuberculosis treatment, in the absence of evidence of disease relapse or the presence of another diagnosis [1]. HIV co-infection is a well-recognised risk factor for this, usually when concomitant anti-retroviral therapy (ART) is started [2] and is termed the Immune Reconstitution Inflammatory Syndrome (IRIS). This is thought to occur when a functioning immune response returns by the action of ART and worsens TB symptoms during treatment [3], or where undiagnosed TB is ‘unmasked’ by ART [4]. TB itself is immunomodulatory, and initiation of TB treatment can also reverse immunedysregulation [5].

In non-HIV positive people, the reported frequency of PR varies widely, but is lower than that seen in HIV positive people [1, 3]. Further, there is some evidence that the immune response differs by ethnic group [6]. Some have considered that PR may occur as a result of pre-existing allergies to medication [7]. Despite advances in our understanding of the immunological profile of patients who develop IRIS, such as recognition of the role of tumour necrosis factor α (TNF- α) and interleukin-6 [8] and other CD4 T-cell activation inflammatory cytokines and chemokines [9], and apart from introduction of ART in HIV-positive individuals, little is known regarding simple to measure baseline predictive factors for PR during anti-tuberculosis therapy [10]. There have been several, small case series from East Asia that have identified risk factors for developing PR. One Taiwanese study of 16 PR cases showed that decreased serum haemoglobin, albumin level, and lymphocyte count were associated with increased risk of pulmonary TB PR compared to over 600 patients who did not develop PR, although in multivariate analysis the differences just reached statistical significance at 5 % [11]. Another study of 16 individuals from Hong Kong found that lower lymphocyte counts at baseline and extrapulmonary disease were identified risk factors compared to 53 patients who did not develop PR [12]. For pleural TB, younger age, high serum albumin level, low proportion of lymphocytes and high proportion of polymorphonuclear cells in pleural fluid was found in 32 patients in South Korea [13]. In another Korean study, 72 patients with pleural TB developed PR, with increased eosinophil count and protein levels in baseline pleural fluid identified as risk factors [14]. Earlier analysis from our group suggested that culture-positive TB predicted PR [15].

To investigate further associations, we conducted a multi-site UK study to determine the relationship between baseline presenting factors, ethnicity and reported PR frequency. Unlike previous studies, here we present patients with a wide range of ethnicities, and explore the impact of the site of active TB disease on PR, and the effect of HIV and the use of ART compared to other possible causes of PR.

## Methods

### Study design

Cohort study and nested case control study.

### Setting

Four UK centres who were a collaborative group of TB partners with a wide mix of ethnicities across their patient populations. Anonymised demographic and clinical data were collated from the Homerton (HH), Royal Free (RFH), and Guy’s and St Thomas’ Hospitals (STH) in London, and Royal Blackburn Hospital (RBH), Blackburn.

### Participants

The cohort comprised all patients with a diagnosis of TB (culture-positive, or culture-negative with a response to TB treatment and no other possible diagnosis) recorded between January 2002 and December 2008 for RFH and HH, January 1999 and December 2008 (Blackburn) and mid-2006 and 2008 (STH, for all patients with electronic notes available). All data were retrospectively collated except for RFH, where half was prospectively collected as part of a study into PR. Baseline data for HIV status and collated ethnicities were recorded for all subjects based on United Kingdom Government’s Office for National Statistics definitions [16]. For example, this grouping includes Black-British, Black-African and Black-Caribbean. PR was diagnosed using the clinical definition detailed above and defined by Breen et al [1]. Possible PR cases were then confirmed by the site specific leads and the principal author on review of patient notes. Two site leads maintained a prospective register of all consecutive TB cases (HH and RBH), including those who has been identified as having a PR event. Two sites reviewed all case notes for evidence of PR (RFH and STH). Patients were followed up for the duration of treatment and reviewed according to local policy, normally one clinic visit after treatment cessation.

**Fig. 1:**
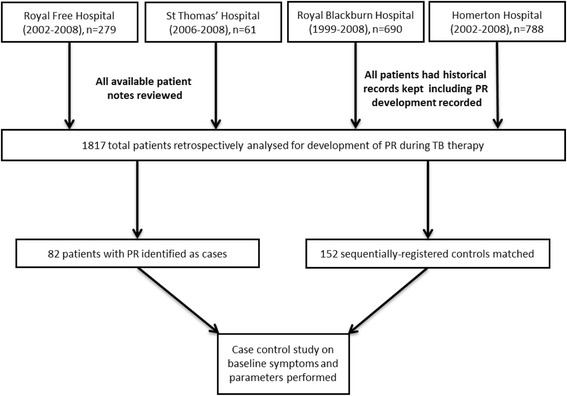
Study population

Those who defaulted follow-up or were found to have another diagnosis were not included in the final assessment. Sequential registration of patients was two to one matched to cases to assess risk factors for PR development, with those who definitely did not have any worsening of symptoms matched to those with a recorded PR. If there were any doubt regarding allocation or uncertainty of symptoms for controls then the subsequent registration was used to ensure that only definitive cases and controls were included in analysis. Figure 1 displays the participant groups.

### Variables

Recorded information included basic demographic details; medical, drug, and allergy history; symptoms at TB diagnosis; physical examination; pre-treatment biochemical, haematological and inflammatory markers; radiographic and histological tests; TB diagnostic tests; and symptoms and evidence of PR.

### Statistical methods

The overall incidence of PR at the four participating centres was calculated, with corresponding 95 % confidence interval (CI). The incidence of PR according to HIV status and ethnic group was also calculated, and compared using a chi-squared test. Finally, the site of disease was summarised according to ethnicity to gain a descriptive understanding of any potential differences.

In the nested case-control study, potential factors associated with PR were examined. Parameters were chosen in advance of analysis: these were demographic factors (age, sex, and ethnicity); immunosuppression (HIV, non-HIV immunosuppression, and the potential immune-modulatory lifestyle factors tobacco and alcohol use); baseline TB disease site, symptoms & microbiology; and baseline blood parameters (total white cell count, lymphocyte count, haemoglobin; albumin and alkaline phosphatase; C-reactive protein and erythrocyte sedimentation rate; lactose dehydrogenase and vitamin D levels).

Analyses were performed using conditional logistic regression to account for matching by calendar time and centre. In order to arrive at a parsimonious model, those potential factors with *p* ≤ 0.1 in univariate analyses were included in the multivariate model, along with a priori assumptions that were brought forward for inclusion (age and sex as routine, and HIV status and TB culture result from our previous findings). No formal adjustments for multiple comparisons were made. Missing data were accounted for with categorical variables, although missing baseline blood tests were not imputed, and analysis limited to those with a blood test available. STROBE guidelines were adhered to for reporting [17].

## Results

### Total patient group

Of 1817 patients treated for TB for whom records were reviewed, 82 (4.5 %, 95 % CI 3.6–5.5 %) subjects with PR were identified. Fifty-five percent of PR cases were female, with a median age of 32 years (IQR 26–41). Twenty-two percent of people with PRs were HIV co-infected (compared to an overall prevalence of 6.9 % of the total 1817 patients). The frequency of PR in the HIV positive group was 14.4 % (95 % CI 8.2–20.6 %) compared to 3.8 % (95 % CI 2.9–4.7) in the HIV negative or untested group (*p* < 0.001).

Table 1 shows the proportion of PR within different racial groups, disaggregated by HIV status. There was no difference in frequency of PR among the different ethnicities in either the HIV positive or HIV negative or unknown groups (*p* = 0.73 and *p* = 0.15). Information on the proportion of patients tested for HIV was available for HH (where testing was performed in 100 % of cases), RFH (testing in 95 %) and STH (92 % cases). At RBH, this was not routinely performed however all patients from injecting drug users or sub-Saharan Africa were tested.

**Table 1:** Ethnicity, HIV status and PR

Ethnicity	Total number	Number HIV positive (%)	Paradoxical reaction number (%; 95 % confidence intervals)		
			Total population	HIV positive	HIV negative/unknown
Black	555	96 (17.3)	32 (5.8; 3.7–7.5)	15 (15.6;8.4–22.9)	17 (3.7; 2.0–5.4)
East Asian	98	5 (5.1)	6 (5.1; 1.4–10.9)	–	6 (6.5; 1.5–11.4)
Mixed Race	11	–	0	–	–
Other	50	4 (13.3)	3 (6.3; 0–12.6)	–	3 (6.5; 0.0–13.7)
South Asian	786	2 (2.5)	33 (5.7; 2.8–5.6)	–	33 (4.2; 2.6–5.6)
White	317	18 (5.7)	8 (5.1; 0.8–4.3)	3 (16.7; 0–33.9)	5 (1.7; 0.2–3.1)
Total	1817	125 (6.9)	82 (4.5; 3.6–5.6)	18 (14.4; 8.2–20.6)	64 (3.8; 2.9–4.7)

Amongst the 82 individuals who experienced PR, the site of disease varied by ethnic group (Table 2). Extra-pulmonary lymph node involvement was found to be significantly more common among South Asians than Blacks adjusted for other ethnicities (*p* = <0.001). Those of Black ethnicity conversely exhibited more pulmonary involvement than people of South Asian and White ethnicity (*p* = 0.045). There was a higher prevalence of systemic symptoms such as fever and sweating amongst those of black and white, compared to South and East Asian ethnicity (40 and 50 % versus 2 and 17 % respectively; *p* = 0.004). HIV co-infection was more common in these PR populations (47 % in the Black PR population and 38 % in the White, none in other groups), though HIV status was not measured in 18 (22 %) of cases. Of note, within the HIV negative population, there was no greater prevalence of baseline systemic symptoms across ethnicities compared to the HIV positive population (*p* = 0.485). Where timing of ART initiation was recorded in HIV positive patients, ART was started prior to or at the time of PR development in 10 cases (56 %) and following TB treatment in 5 cases (28 %). 3 HIV positive patients were not recorded as receiving ART (17 %).

**Table 2:** Site of PR by ethnicity

Site of PR^a^	Number of patients by ethnicity (percentage of total ethnic group with PR)				
	Black	East Asian	Other	South Asian	White
Total	32 (100 %)	6 (100 %)	3 (100 %)	33 (100 %)	8 (100 %)
Chest^b^	15 (47 %)	1 (17 %)	–	6 (18 %)	1 (13 %)
Systemic Symptoms^c^	13 (40 %)	1 (17 %)	–	2 (6 %)	4 (50 %)
Abdominal	6 (19 %)	–	–	–	1 (13 %)
Brain	2 (6 %)	–	–	–	–
Peripheral lymph nodes	8 (25 %)	4 (67 %)	3 (100 %)	25 (76 %)	4 (50 %)
Other	2 (6 %)	1 (17 %)	1 (33 %)	1 (3 %)	3 (38 %)

^a^PR may occur at more than one site for each patient, so percentages do not sum to 100 %

^b^Pulmonary, pleural, and mediastinal lymph nodes

^c^Systemic symptoms are persistent fever and persistent night sweats

The majority of PR occurred at the presenting site of TB disease (81; 88 %), with 91 % (84) having an exacerbation of pre-diagnosis symptoms. The median time of onset of PR was at week 4.5 (IQR 3–11, maximum of 150 weeks in one patient who represented after treatment completion). The median duration of PR was 4 weeks (range 1–46 weeks). Forty-five percent (41) received treatment with steroids.

### Case control analysis

In total 152 controls were selected for the 82 cases; 12 cases could only be matched to one control, and 70 cases were matched to two controls. Table 3 provides information on selected demographics, baseline inflammatory markers, allergies reported to medication, TB smear and culture, and presenting symptoms in the PR and non-PR groups. There were no observed differences in age or sex between cases or controls. Pulmonary disease was more common as the presenting site of initial disease in controls (63 % versus 38 %), and peripheral lymph nodes more frequent in PRs (42 % versus 3 %; *p* < 0.001). There were somewhat more HIV-positive individuals in the PR group (22 % versus 16 %; *p* = 0.41).

**Table 3:** Univariate analysis of baseline variables at TB diagnosis in case control cohort

Variable (*N* = 234 total unless otherwise stated)		PR (%) (*N* = 82)	Matched control without PR (*N* = 152)	*P* value^*^
Demographics^a^				
Age (Mean)		33.7	37.0	0.162
Sex (%)	Male	72 (45 %)	72 (47 %)	–
	Female	45 (55 %)	80 (53 %)	0.770
Ethnicity (%)	White	8 (10 %)	26 (17 %)	–
	Black	32 (39 %)	65 (43 %)	–
	East Asian	6 (7 %)	9 (6 %)	–
	South Asian	33 (40 %)	46 (30 %)	–
	Other	3 (4 %)	6 (4 %)	0.663
Baseline TB disease site, symptoms & microbiology				
Site (%)	Chest ^b^	29 (35 %)	96 (63 %)	–
	Abdominal	2 (2 %)	4 (3 %)	–
	Brain	2 (2 %)	5 (3 %)	–
	Peripheral lymph nodes	34 (41 %)	4 (3 %)	–
	Other or mixed sites	15 (18 %)	43 (28 %)	<0.001
Systemic Symptoms (%)^c^ (with weight loss)	Yes	23 (28 %)	45 (30 %)	–
	No	54 (66 %)	101 (66 %)	–
	Not recorded	5 (6 %)	6 (4 %)	0.901
Systemic Symptoms (%)^c^ (without weight loss)	Yes	27 (33 %)	64 (42 %)	–
	No	50 (61 %)	82 (54 %)	–
	Not recorded	5 (6 %)	6 (4 %)	0.332
Acid Fast Bacilli (%)	Negative	47 (57 %)	88 (58 %)	–
	Positive	25 (30 %)	42 (28 %)	–
	Not performed	10 (12 %)	22 (14 %)	0.770
*M. tuberculosis* NAAT (%)	Negative	8 (10 %)	9 (6 %)	–
	Positive	20 (24 %)	24 (16 %)	–
	Not performed	54 (66 %)	119 (78 %)	0.007
*M. tuberculosis* Culture (%)	Negative	13 (16 %)	37 (24 %)	–
	Positive	62 (76 %)	104 (68 %)	–
	Not performed	7 (9 %)	11 (7 %)	0.256
Immune system functioning				
HIV status (%)	Negative	46 (56 %)	101 (66 %)	–
	Positive	18 (22 %)	25 (16 %)	–
	Not recorded	18 (22 %)	26 (17 %)	0.413
Drug allergies (%)	No	34 (41 %)	60 (39 %)	–
	Yes	2 (2 %)	2 (1 %)	–
	Not recorded	46 (56 %)	90 (59 %)	0.688
Immunosuppression (%)^d^	No	80 (98 %)	130 (8^%)	–
	Yes	1 (1 %)	17 (11 %)	–
	Not recorded	1 (1 %)	5 (3 %)	0.004
Potential immune–modulating lifestyle factors				
Tobacco use (%)	No	60 (73 %)	97 (64 %)	–
	Yes	10 (12 %)	37 (24 %)	–
	Not recorded	12 (15 %)	18 (12 %)	0.101
Alcohol use (%)	No	59 (72 %)	85 (56 %)	–
	Yes	8 (10 %)	34 (22 %)	–
	Not recorded	15 (18 %)	33 (22 %)	0.007
Geometric mean (range) baseline blood test results^e^ (units, number of patients with variable data)				
White blood cell count (cells/μL, *n* = 229)		7.2 (3.1–16.1)	7.2 (2.1–18.4)	0.953
Lymphocyte Count (cells/μL, *n* = 229)		1.6 (0.4–4.2)	1.6 (0.3–6.5)	0.855
Haemoglobin (g/dL, *n* = 230)		11.7 (7.7–16.0)	12.0 (7.1–17.3)	0.285
Albumin (g/dL, *n* = 225)		38 (16–77)	37 (9–50)	0.501
Alkaline Phosphatase (U/L, *n* = 225)		134 (44–823)	123 (14–1074)	0.627
C-Reactive Protein (mg/L, *n* = 203)		62 (1–280)	65 (1–396)	0.939
Erythrocyte Sedimentation Rate (mm/h, *n* = 139)		56 (6–132)	46 (2–129)	0.017
Vitamin D Levels (ng/mL, *n* = 85)		27.1 (8.6–79.4)	30.9 (6.3–168.0)	0.764
Lactate Dehydrogenase (U/L, n = 99)		499 (294–1156)	523 (246–2127)	0.699

^*^ *P* values calculated using Wald tests in conditional logistic regression

^a^Demographic information available for all patients

^b^Pulmonary, pleural, and mediastinal lymph nodes

^c^Systemic symptoms are persistent fever and persistent night sweats

^d^Non-HIV immunosuppressive state such as diabetes mellitus, cancer, chronic renal failure or pregnancy

^e^Log means were used for analysis to approximate normal distributions for skewed tails

A positive mycobacterial culture and Nucleic Acid Amplification Technique (NAAT), lack of tobacco and alcohol use, a raised baseline ESR, the absence of non-HIV immunosuppression (defined here as diabetes mellitus, cancer, chronic renal failure or pregnancy), and presence of initial site of disease at extrathoracic lymph nodes were associated with development of PR in univariate analysis. In multivariate analysis (Table 4), presence of baseline LN disease, TB culture positivity, HIV positive status, and presence of non-HIV immunosuppression were all associated with the development of PR at a 5 % level of significance.

**Table 4:** Multivariate analysis of factors associated with development of PR: results from conditional logistic regression model

Variable		Odds ratio (95 % CI)	*P* value
Age		0.98 (0.95–1.02) per increasing year	0.409
Sex		0.57 (0.19–1.67) for females	0.302
Site	Chest	1.00	–
	Abdominal	8.11 (0.18–356.01)	–
	Brain	2.22 (0.22–23.30)	–
	Peripheral lymph nodes	64.33 (9.60–431.25)	–
	Other or mixed sites	1.23 (0.41–3.74)	<0.001
HIV Status	Negative	1.00	–
	Positive	5.05 (1.28–19.85)	–
	Not recorded	0.50 (0.02–14.97)	0.028
Immunosuppression	No	1.00	–
	Yes	0.01 (0.00–0.27)	–
	Not recorded	0.13 (0.00–0.90)	0.002
Tobacco use	No	1.00	–
	Yes	0.71 (0.17–2.85)	–
	Not recorded	3.36 (0.39–21.04)	0.462
Alcohol use	No	1.00	–
	Yes	0.21 (0.04–1.01)	–
	Not recorded	0.01 (0.01–0.56)	0.009
ESR^a^	Mean (mm/h)	Could not fit within model	–
TB Diagnosis	% NAAT negative	1.00	–
	% NAAT positive	1.23 (0.11–12.63)	–
	% NAAT not performed	0.10 (0.01–1.11)	0.009
	% culture negative	1.00	–
	% culture positive	6.87 (1.31–36.04)	–
	% culture not performed	3.81 (0.66–22.14)	0.045

^a^ESR was only performed on a limited number of patients (*n* = 139, only 97 of which could be used for case control analysis): it was unable to be included in the model due to small numbers. In univariate analysis a 1-log increase in ESR was associated with a 4.43 increased odds of developing PR (95 % CI 1.30–15.1); this increased to 4.78 (1.29–17.72) when adjusted for HIV status

The association with HIV was confirmed in multivariate analysis, where the odds ratio of developing PR were 5.05 (95 % CI 1.28–19.85, *p* = 0.028) compared to those without known HIV infection.

Lymph node disease was associated with a 64.33-fold increased odds of having a PR event (95 % CI 9.60–431.25, *p* < 0.001). A positive TB culture increased the odds of developing PR 6.87 times (95 % CI 1.31–36.04, *p* = 0.045). Though NAAT showed an association, the greatest percentage difference in the non-PR and PR groups was in those who did not have a test performed. Finally, non-HIV immunosuppression reduced the risk of PR significantly (aOR 0.01; 95 % CI 0.00–0.27, *p* = 0.002).

Baseline ESR could not be fitted within the multivariate model due to the limited numbers of people in whom this was measured (*n* = 48 in cases, *n* = 91 in controls, only 97 of which were able to be used in matched case control analysis; see Table 3). Sensitivity analysis dividing ESR into either three or four categories confirmed increasing odds of PR with increasing ESR, but again this could not be fitted into multivariate analysis due to small numbers. ESR is often moderately elevated in HIV infection [18]. In univariate analysis a 1-log increase in ESR was associated with a 4.43 increased odds of developing PR (95 % CI 1.30–15.1); this association remained in sensitivity analysis when adjusted for HIV status (aOR 4.78; 1.29–17.72, *p* = 0.019).

## Discussion

Within our total population, we find that HIV is strongly associated with development of PR, mainly in the context of ART initiation within the case control cohort (55 % started ART before or at time of PR development). The observed frequency of 14.4 % within the HIV positive group is similar to that of 15.7 % reported in a meta-analysis of 3500 HIV co-infected patients [19]. In the HIV negative or unknown group our observed frequency of 3.8 % is also in line with published observations (ranging from 2 % in all patients to 25 % in extra-pulmonary tuberculosis) [1, 2, 20]. The long length of time to PR onset that can occur (up to a maximum of 150 weeks in our cohort) has been previously recognised [21], and our observed median time to onset is similar to other general studies [1], though shorter than those looking solely at extra-pulmonary PR [20].

We observed an association between the location of the PR site and ethnicity, although the overall rate of PR did not differ between ethnic groups. This likely arises because baseline disease site (which does appear to have a racial origin) heavily influences the site of PR: our Black and White study populations had more pulmonary disease compared to those of South Asian ethnicity, in whom lymphadenitis predominates. This is in keeping with other reports [22].

Our findings suggest that peripheral lymph node disease greatly predisposes to PR; despite large confidence intervals, the increased risk is pronounced, in the magnitude of sixty-fold increased odds in multivariate analysis. This has been previously documented as a likely site of IRIS. In the third British Thoracic Society lymph node study, carried out in a predominantly South Asian cohort between 1987–89 i.e. largely without HIV co-infection, new lymphadenopathy was reported in 16 % in the first three months of treatment [23], and new nodes or sinuses in 7–12 % after treatment completion [24]. We cannot exclude ascertainment bias as a possible cause of this; and PR at other less easily-identifiable locations might be under-reported in the absence of systemic symptoms justifying further investigations. This potential confounding supports the need for an international consensus to ensure that all potential paradoxical reactions are appropriately identified and recorded to improve epidemiology and surveillance [25].

As reported with TB meningitis IRIS in South Africa [26] and disseminated TB in a Malaysian HIV cohort [27], the increased potential for PR in people with culture positive tuberculosis confirms that underlying host mycobacterial burden appears important in the development of PR, though some culture negative disease may not in fact be TB but rather another diagnosis. Therefore culture positive disease could be over-represented artefactually in analysis. It should be noted, however, that we also found PRs in apparent low mycobacterial burden disease (as 16 % were culture negative at baseline).

There was evidence that the combined group of non-HIV immunosuppression including diabetes was associated with reduced odds of developing PR. Whilst the numbers in this heterogeneous population are small (*n* = 18, seven of whom were diabetic, and none were using or recently withdrawn from biologic treatments) with wide confidence intervals, PR has occurred in oncology patients receiving granulocyte colony-stimulating factor post chemotherapy, though not when functionally impaired [28], and on TNF-α antagonist withdrawal [29]. We are unaware of a reported association between PR and diabetes, and this subgroup should be explored in larger datasets.

Although numbers were small, we observed a trend towards a lower risk of developing PR with increased alcohol use (aOR 0.21; 0.04–1.01; group categorical *p* value of 0.009, alcohol use versus no alcohol, *p* = 0.051). This, again, would be interesting to further evaluate in a larger study.

Recent studies have suggested that baseline C-Reactive Protein (CRP) is associated with the risk of developing PR in HIV co-infected individuals [30, 31]. This requires confirmation in a larger longitudinal study. From our data it appears that the Erythrocyte Sedimentation rate (ESR), which has also been shown to be raised at baseline in TB [32] may be a useful predictive marker or PR. Given the limited data on ESR within our cohort, this will require further work to determine whether an association exists.

### Strengths and limitations

There are several limitations of this study. Ascertainment of development of PR was collected retrospectively at two centres, and therefore we cannot be certain all true PR cases were identified, although case notes were reviewed thoroughly by both the site PI and the study lead author. Furthermore, the case control nature of the primary analysis limits the potential biases caused by this. Selection bias may result in identification of the more severe cases that required treatment. In particular for some blood measures, not all tests were performed in all patients. For the total population, HIV testing was not performed in every patient and although testing was routinely offered at the diagnosis of TB, there is the potential for HIV status to be assessed more assiduously in diagnosed or suspected PR cases. The one setting where this was not performed regularly has a low overall HIV prevalence. In our study, where known, overall testing rates for HIV were above 90 %. Given the relatively small numbers of PR, some confidence intervals are wide in multivariate analysis. However the large baseline denominator from whom the controls were drawn had a broad representation of age, ethnicity, sex, and baseline disease. We believe our findings highlight areas for future study, and can be generalised to other population settings.

## Conclusions

Overall, this retrospective case control study highlights the known association between HIV and PR during TB treatment. It did not succeed in identifying any clear additional positive or negative risk factors apart from possible mycobacterial burden and non-HIV immunosuppression; or confirm previous blood parameters from smaller studies. This demonstrates the difficulties in predicting PR using routinely available demographic details, clinical symptoms or biochemical markers. It also highlights the apparent frequency of PR when it occurs at visibly apparent sites such as peripheral lymph nodes. To determine whether PR risk stratification, based on easy to measure variables at initial presentation is possible, greater numbers of prospectively-gathered PR cases need to be assessed. A positive result would enable people at highest risk to be monitored carefully, with appropriate therapy started to treat, or even prevent PR.

## Abbreviations

AFB: Acid Fast Bacilli
ART: Anti-retroviral therapy
CRP: C-reactive protein
ESR: Erythrocyte sedimentation rate
HIV: Human immunodeficiency virus
IQR: Inter-quartile range
IRIS: Immune Reconstitution Inflammatory Syndrome
MTB: *Mycobacterium tuberculosis*
NAAT: Nucleic Acid Amplification Technique
PR: Paradoxical reaction
TB: Tuberculosis
